# Healthcare provider cost of antimicrobial resistance in two teaching hospitals in Ghana

**DOI:** 10.1093/heapol/czad114

**Published:** 2023-12-04

**Authors:** Evans Otieku, Joergen Anders Lindholm Kurtzhals, Ama Pokuaa Fenny, Alex Owusu Ofori, Appiah-Korang Labi, Ulrika Enemark

**Affiliations:** Economics Division, Institute of Statistical, Social and Economic Research (ISSER), University of Ghana, P.O. Box LG 74, Accra 233, Ghana; Department of Public Health, Aarhus University, Batholins Alle 1, Building No. 1261, Aarhus 8000, Denmark; Centre for Medical Parasitology, Department of Immunology and Microbiology, University of Copenhagen, Copenhagen 1165, Denmark; Department of Clinical Microbiology, Rigshospitalet, Copenhagen University Hospital, Copenhagen 1165, Denmark; Economics Division, Institute of Statistical, Social and Economic Research (ISSER), University of Ghana, P.O. Box LG 74, Accra 233, Ghana; Laboratory Services Directorate, Komfo Anokye Teaching Hospital, Kumasi 233, Ghana; Department of Clinical Microbiology, Korle-Bu Teaching Hospital, School of Medicine and Dentistry, Kwame Nkrumah University of Science and Technology, Kumasi 233, Ghana; Department of Medical Microbiology, Korle-Bu Teaching Hospita, University of Ghana Medical School, Accra 233, Ghana; Department of Public Health, Aarhus University, Batholins Alle 1, Building No. 1261, Aarhus 8000, Denmark

**Keywords:** Antimicrobial resistance, healthcare provider, economic cost, policymaking

## Abstract

Understanding the healthcare provider costs of antimicrobial resistance (AMR) in lower-middle-income countries would motivate healthcare facilities to prioritize reducing the AMR burden. This study evaluates the extra length of stay and the associated healthcare provider costs due to AMR to estimate the potential economic benefits of AMR prevention strategies. We combined data from a parallel cohort study with administrative data from the participating hospitals. The parallel cohort study prospectively matched a cohort of patients with bloodstream infections caused by third-generation cephalosporin-resistant enterobacteria and methicillin-resistant *Staphylococcus aureus* (AMR cohort) with two control arms: patients infected with similar susceptible bacteria and a cohort of uninfected controls. Data collection took place from June to December 2021. We calculated the cost using aggregated micro-costing and step-down costing approaches and converted costs into purchasing power parity in international US dollars, adjusting for surviving patients, bacterial species and cost centres. We found that the AMR cohort spent a mean of 4.2 extra days (95% CI: 3.7–4.7) at Hospital 1 and 5.5 extra days (95% CI: 5.1–5.9) at Hospital 2 compared with the susceptible cohort. This corresponds to an estimated mean extra cost of $823 (95% CI: 812–863) and $946 (95% CI: US$929–US$964) per admission, respectively. For both hospitals, the estimated mean annual extra cost attributable to AMR was approximately US$650 000. The cost varies by organism and type of resistance expressed. The result calls for prioritization of interventions to mitigate the spread of AMR in Ghana.

Key messages

- From the provider’s perspective, we evaluated the economic cost of antimicrobial resistance (AMR) in sepsis patients.
- We used quality hybrid data that make our findings reliable in the Ghanaian context and show that AMR is associated with increased length of stay and cost to healthcare providers.
- Our findings make a strong case for investment in AMR interventions by policymakers and hospital managers to prevent extra provider costs due to AMR.

## Introduction

The rise in antimicrobial resistance (AMR) is a global public health concern (Smith and Coast, 2002; World Health Organization, 2020). A recent global study estimated that 4.95 million deaths in 2019 were associated with bacterial AMR (Antimicrobial Resistance (AMR) Collaborators, 2022). Besides mortality, AMR has been associated with prolonged morbidity, length of hospital stay (LOS) and associated costs (Founou *et al.*, 2017; Dadgostar, 2019).

The micro- and macro-economic implications of AMR are mounting. By 2050, AMR is projected to cause a 3.8% decline in the global gross domestic product, and the impact is estimated to be higher in low- and middle-income countries (LMICs) (The World Bank, 2017). Over the same period, the annual global healthcare costs due to AMR may increase from US$300 billion to US$1 trillion (The World Bank, 2017; Ahmad and Khan, 2019).

Evidence shows that the inappropriate antibiotic use increases the risks of AMR (World Health Organization, 2015). Both supply and demand factors contribute to the misuse of antibiotics and the consequent spread of AMR (Labi *et al.*, 2018; Sakeena *et al.*, 2018; D’Arcy *et al.*, 2021). On the supply side, inappropriate antibiotic prescribing by doctors may be influenced by short- and long-term financial interests (Martinez-Nadal *et al.*, 2020; Kadri *et al.*, 2021). On the demand side, increasing income may increase demand for antibiotics (Lin *et al.*, 2020). Thus, rising income levels in Ghana may have a corresponding effect on health service use, including increased demand for medications like antibiotics leading to a spread of AMR.

AMR creates a hydra-headed problem for healthcare providers in LMIC settings, where resources are limited. On the one hand, the capacity of hospitals to admit more patients needing critical and emergency care may be constrained by limited bed capacity as patients with AMR occupy beds. This may result in morbidity and mortality that could have been avoided if AMR was prevented (Majeed *et al.*, 2012; Rojas-García *et al.*, 2018). At the same time, healthcare providers may have to commit limited resources to treat patients with AMR infections.

Recent findings show very high prevalence of AMR infections in Ghana. In a sentinel site study, 88% of Gram-negative bacteria isolated from bloodstream infections were multi-drug resistant and 56–78% of the organisms were resistant to third-generation cephalosporins (3GCs) (Donkor *et al.*, 2023). In a study of surgical site infections, 86% of *Eschericia coli* were multi-drug resistant and 24% of *Staphylococcus aureus* were methicillin resistant (Bediako-Bowan *et al.*, 2020). Several reasons account for the spread of AMR in Ghana, including the lack of adherence to antibiotic prescribing protocol by health professionals (Sumaila and Tabong, 2018; Owusu *et al.*, 2022). At the same time, systematic monitoring is not taking place concurrently at all levels of care because most hospitals do not have well-equipped laboratory for AMR surveillance. Ghana has a national AMR action plan to respond to these and other AMR-related challenges. However, progress regarding the plan implementation is slow and compounded by limited resource allocation (Hein *et al.*, 2022). In line with Ghana’s national action plan, there is a need to strengthen the motivation of key stakeholders at various levels (individual prescribers, hospital managers and government stewardship) to implement this plan.

Therefore, awareness of AMR costs to the health system and the government may stimulate a more conservative approach to antibiotic prescription and strict adherence to the prescription protocol. In addition, evaluation of the health system costs related to AMR may stimulate an interest in investing in AMR interventions, including enforcement of regulations to counter demand-side factors contributing to the spread of AMR (Roope *et al.*, 2019; Jit *et al.*, 2020).

The present study adds to previous studies in which we have evaluated the patient costs of AMR and the behaviours predicting antibiotic use (Otieku *et al*., 2023a; 2023b). Thus, in this study, we aim to investigate whether healthcare providers incur additional costs for treating AMR infections compared with a cohort of susceptible AMR infections and uninfected cohorts.

## Methods

### Design

We combined data of patients from a parallel cohort study (PCS) by the authors (Otieku *et al.*, 2023) with administrative data from the participating hospitals to evaluate the healthcare provider cost (HPC) of AMR in Ghana. Patients in the PCS were followed up for 30 days after diagnosis of a positive blood culture until discharged from the hospital or death, whichever occurred first. See the Population and Sampling section for details on inclusion and matching criteria for the PCS. Reporting followed the relevant sections of the Consolidated Health Economic Evaluation Reporting Standard checklist (Husereau *et al.*, 2022). Consent to provide administrative data was granted by administrative heads of the participating hospitals.

### Setting

Study settings were two public teaching hospitals in Ghana, an LMIC in West Africa. Conditions for selecting the hospitals were as follows: (1) the feasibility to complete the PCS data collection from June to December 2021, and (2) together, the hospitals account for 60% of the total bed capacities of public tertiary hospitals in Ghana and were comparable in terms of population coverage and hospital resources, including microbiology laboratories capable of processing an average of 4000 blood culture examinations and AMR susceptibility analyses yearly.

### Population and sampling

The PCS population included all inpatients with suspected bloodstream infection (BSI) who had blood cultures performed at the central microbiology laboratories of the participating hospitals during participant recruitment from June to December 2021. A sample of the study population with positive blood cultures of *E. coli* or *Klebsiella* spp., resistant to 3GC, or with methicillin-resistant *S. aureus*(MRSA) constituted the AMR cohort. In short, the independent exposure of interest was bloodstream infections due to resistant bacteria compared to susceptible bacteria. However, we also evaluated the magnitude of the potential cost savings if bloodstream infections are avoided entirely by comparing the provider cost between the AMR and uninfected cohorts. Patients lost to follow-up with incomplete data or who declined participation were excluded. We determined a required sample size of 400 (95% CI: 322–478) for the AMR cohort based on a 50% population proportion at a 5% error margin and 95% CI (*z*-score: 1.96). We matched the participants in the AMR cohort with two control arms, that is, patients with the same bacterial infections non-resistant to 3GC or methicillin (susceptible cohort) and uninfected patients (uninfected cohort). Briefly, for each new AMR patient included, we sought out one susceptible and one uninfected patient based on age group (±5 years), admission ward, gender and bacteraemia type for susceptible AMR patients (Otieku *et al.*, 2023).

### Data sources

#### Patient-level data

As described in the study on the patient cost of AMR (Otieku *et al.*, 2023), denominator data were collected in person from patients by trained hospital staff (intern nurses) using a computer-assisted personal interviewing tool embedded with a validated data collection protocol design with CS Pro version 7.6.0 software. The relevant data included the LOS, ward of admission and hospital resource use, including doctors’ time consumed, measured in minutes, which was available in the patients’ hospital folder.

#### Hospital-level data

We obtained administrative data on hospital resource use and expenditures for the 2021 financial year. The resource use data included staffing, number of admissions and the average LOS for the total number of admissions in 2021. The expenditure data comprised staff remuneration and allowances, annualized capital expenditure (assets) and the expenses on goods and services, defined as the sum of the expenses on medical supplies, utilities and maintenance services. The data were extracted from the hospitals’ unpublished annual financial reports.

### Outcomes

Primary outcome measures were the extra length of stay and associated hospital costs attributed to bloodstream AMR infections. A secondary outcome measure was the amount of time doctors spent treating patients with bloodstream AMR infection.

### Measurement of outcomes

#### Length of stay

We equated the extra day(s) healthcare providers spent treating bloodstream AMR infections to AMR-attributable LOS, measured as the sum of inpatient days, adjusted for hospital stays before a diagnosis of bacteraemia to avoid lead-time bias that may contribute to overestimation of LOS (Nelson *et al.*, 2015). Doctors’ time loss due to AMR was measured in minutes and obtained from electronic patients’ hospital records.

#### Costing approaches

We performed primary and secondary cost analyses using gross costing approaches (Hellebo *et al.*, 2021; Ifeanyichi *et al.*, 2022). Gross costing encompasses the summation of detailed cost accounting identities of recurrent and annualized fixed expenses incurred by the hospitals and obtained from the hospital’s annual financial reports. We assumed that all hospital expenditures were directly or indirectly related to the cost of AMR care. Therefore, all recurrent and annualized fixed expenditures were considered. The recurrent expenditures included staff remuneration and allowances that considered the overhead gross salaries and allowances paid through central government payroll for the established staff and from the hospital internally generated funds for the non-established staff, multiplied by the quantity of staff in each rank (see Table S1). Others comprised the total expenses on medical consumables, utilities (i.e. electricity, water and sewage), medical waste management, asset maintenance service, transportation and communication service charges, etc. The annualized capital costs were calculated from an asset register by accredited professional valuers contracted by the hospitals. The asset register comprised inventories of all available assets, including buildings, furniture/fittings and equipment as recommended in the WHO’s manual for estimating hospital costs (Shepard *et al.*, 2000).

### Analysis

Both statistical and cost analyses were performed using STATA version 14 software and Microsoft Excel.

#### Statistical analysis

From the PCS data, we compared background characteristics of patient groups by stratifications into surviving patients, bacteria type and cost centre using chi-squared statistics. We calculated AMR-attributable LOS using *t*-test statistics for two cohorts at a time and a negative binomial regression with 95% CIs.

#### Cost analysis

In the primary analysis, we divided the total hospital expenditure by the total hospital bed days in 2021 (Equation 1) and multiplied that by the extra LOS attributable to AMR to estimate the HPC associated with AMR (Equation 2):


(1) $${\rm HPC}_{inpatient}\, = \,\left[ {Total{\ }expenditure/Total{\ }bed{\ }days} \right]$$

where ‘HPC_inpatient_’ is the HPC per inpatient, ‘*Total expenditures*’ equal the aggregated annual hospital expenditure on staff remuneration/allowances, consumables and annualized costs of assets (capital expenditure), while ‘*Total bed days*’ were derived by multiplying the total number of inpatients in each hospital in 2021 by the mean LOS obtained from the unpublished hospital’s annual report. This implicitly assumes that on average, the number of resources used such as laboratory/diagnostic services, ambulatory visits, etc., is the same for all patients.


(2) $${\rm HPC}_{AMR}\, = \,\sum \left[ {\rm HPC}_{inpatient}\, \times \,Extra{\ }bed{\ }days{\ }due{\ }to{\ }AMR \right]$$

where ‘HPC_*AMR*_’ denotes HPC incurred on each patient due to AMR, while the ‘*Extra bed days due to AMR*’ is the AMR-attributable LOS calculated as the mean difference in LOS between the AMR and susceptible cohorts.

The results were stratified by hospital, bacteraemia type and surviving patients. Adjusting the costs for surviving patients was relevant because they tend to have longer LOS and use more hospital resources compared to patients who died before the end of the 30-day follow-up. For each cost centre, we evaluated the percentage change in the endpoint costs as the cost difference between the crude and secondary estimated mean costs with 95% CI.

#### Secondary cost analysis

In the step-down costing analysis, the empirical rule of thumb for evaluating hospital costs from the provider perspective is that the endpoint costs may differ by centre, unit or department due to differences in resource allocation and consumption such as staffing, medical supplies/consumables and assets. Therefore, we performed a secondary analysis of the centre-specific costs of AMR by multiplying the estimated provider cost per bed day by the estimated LOS due to AMR for each cost centre/department. The analysis was, in essence, a check of the robustness of the crude estimates in the primary analysis (Ifeanyichi *et al.*, 2022). Detailed assumptions underpinning the cost allocations are described in Tables S2–S5 (see Supplementary File). Briefly, we captured only expenses related to services delivered by the cost centres for purposes of precision. For example, the pooled expenditure incurred by the Department of Child Health was split into three, that is, paediatric, neonatal intensive care and paediatric intensive care using staff speciality, bed capacity/admissions, assets and the goods and services consumed as cost allocation factors. At the Surgical Department, costs of providing services such as neurosurgery, paediatric surgery, urology and allied surgery (dental, eye, ear, nose and throat) were excluded because no AMR cases were recorded at these units. We classified the directorate of laboratory service as rendering multicentre-purpose services by providing access to patients from all clinical departments in the hospital. The distribution of the shared cost of laboratory service use by patients between departments considered two approaches. The best approach is using the actual laboratory resources consumed by patients from all clinical departments, considering variations in bed capacity and duration of admission. However, data on actual resource use was unavailable at the time of the data collection due to shift from paper-based to digital-based data processing system. Information obtained from personnel at the laboratory suggests that there is no perceived significant difference in the annual resource use between departments as smaller units tend to have a higher intensity of laboratory tests (Table S2). Therefore, we used the alternative approach of dividing the annual laboratory directorate cost by the number of clinical departments and allocated the same amount to each cost centre (Ifeanyichi *et al.*, 2022). Staff cost considered the number of staff rendering clinical, administrative and auxiliary services multiplied by the average unit cost of personnel in each cadre/speciality at the cost centre. In the child health department in Hospital 2, some resident doctors rotate between the neonatal intensive care unit, paediatric unit and the paediatric intensive care unit. The head count of rotation staff was counted only once for the unit where they had their employment. We assumed that the time allocation between units nets out as staff from each unit help each other out. Again, personnel costs excluded the salaries and allowances of personnel from other institutions providing collaborative short-term clinical support services at the hospitals as part of an exchange programme.

All costs were converted from the local currency (Ghana cedis) to 2021 purchasing power parity (PPP) in international US dollars using a standard web-based calculator (Ian *et al.*, 2010) and rounded up to the nearest whole number.

## Results

### Descriptive statistics

We recruited 219 patients with AMR from 3150 blood cultures performed at Hospital 1 and 207 AMR patients from 2602 blood cultures at Hospital 2. At Hospital 1, we lost 5.0% (11 patients) to follow-up, and 1.4% (3 patients) refused consent. Likewise, we lost 3.4% (7 patients) to follow-up, and one person refused participation at Hospital 2, representing a completion rate of ∼94% at Hospital 1 and 96% at Hospital 2. We matched the AMR patients to the same number of patients with no BSI (uninfected cohort) and about a third of patients with BSI not resistant to screening antibiotics (susceptible cohort, *n* = 79 at Hospital 1; *n* = 73 at Hospital 2). The low number of patients in the susceptible cohort was due to the high prevalence of AMR. Thus, a suitable susceptible control could only be found for less than half of AMR patients. The three cohorts had similar distributions of gender, age, bacterial isolate (in the infected groups), treatment ward and comorbidity (Table 1). The survival among infected patients was significantly higher in the susceptible than in the AMR cohort (*P* = 0.02). Due to the imperfect age criterion for matching, 11 patients in the AMR cohort had more severe diseases than their matches in the susceptible and uninfected groups.

**Table 1.** Participant characteristics

	Overall		Hospital 1			Hospital 2		
Variable	Category	*n* (%)	AMR cohort	Susceptible cohort	Uninfected cohort	AMR cohort	Susceptible cohort	Uninfected cohort
Age groups (years)	0–4 5–14 15–24 25–34 35–44 45–54 ≥55 Total	232 (24.2) 121 (12.6) 49 (5.1) 54 (5.6) 78 (8.1) 122 (12.7) 304 (31.7) 960 (100.0)	38 (18.5) 24 (11.7) 10 (4.9) 16 (7.8) 18 (8.8) 27 (13.2) 72 (35.1) 205 (100.0)	10 (12.6) 14 (17.7) 4 (5.1) 4 (5.1) 7 (8.9) 10 (12.6) 30 (38.0) 79 (100.00)	30 (14.6) 28 (13.7) 9 (4.4) 16 (7.8) 17 (8.3) 32 (15.6) 73 (35.6) 205 (100.0)	76 (38.2) 20 (10.1) 11 (5.5) 8 (4.0) 13 (6.5) 17 (8.5) 54 (27.2) 199 (100.0)	30 (41.1) 9 (12.3) 3 (4.1) 3 (4.1) 3 (4.1) 7 (9.6) 18 (24.7) 73 (100.0)	66 (33.4) 27 (13.6) 12 (6.0) 6 (3.0) 13 (6.5) 20 (10.1) 55 (27.6) 199 (100.0)
Gender	Female Male Total	496 (51.7) 464 (48.3) 960 (100.0)	101 (49.3) 104 (50.7) 205 (100.0)	33 (41.8) 46 (58.2) 79 (100.0)	101 (49.3) 104 (50.7) 205 (100.0)	109 (54.8) 90 (45.2) 199 (100.0)	43 (58.9) 30 (41.1) 73 (100.0)	109 (54.8) 90 (45.2) 199 (100.0)
Bacteria isolate	*E. coli* *Klebsiella* spp. *S. aureus* Total	178 (32.0) 178 (32.0) 200 (36.0) 556 (100.0)	48 (23.4) 65 (31.7) 92 (44.9) 205 (100.0)	19 (24.1) 25 (31.6) 35 (44.3) 79 (100.0)		86 (43.2) 58 (29.2) 55 (27.6) 199 (100.0)	25 (34.2) 30 (41.1) 18 (24.7) 73 (100.0)	
Treatment ward	Emergency Maternity Medical^1^ NICU PICU Paediatric Surgical^2^ Total	90 (9.4) 10 (1.0) 444 (46.3) 28 (2.9) 50 (5.2) 265 (27.6) 73 (7.6) 960 (100.0)	25 (12.2) 3 (1.5) 107 (52.2) 47 (22.9) 23 (11.2) 205 (100.0)	11 (13.9) 40 (50.6) 21 (26.6) 7 (8.9) 79 (100.0)	25 (12.2) 3 (1.5) 107 (52.2) 47 (22.9) 23 (11.2) 205 (100.0)	12 (6.0) 2 (1.0) 81 (40.7) 11 (5.5) 22 (11.1) 62 (31.2) 9 (4.5) 199 (100.0)	5 (6.9) 28 (38.4) 6 (8.2) 6 (8.2) 26 (35.6) 2 (2.7) 73 (100.0)	12 (6.0) 2 (1.0) 81 (40.7) 11 (5.5) 22 (11.1) 62 (31.2) 9 (4.5) 199 (100.0)
Health insurance	(Yes)	843 (87.8)	169 (82.4)	69 (87.3)	178 (86.8)	168 (84.4)	71 (97.3)	188 (94.5)
Mortality	(Yes)	80 (8.3)	29 (14.2)	6 (7.6)	8 (3.9)	33 (16.2)	2 (2.7)	2 (1.01)
Comorbidity^3^	(Yes)	517 (93.0)	191 (93.2)	74 (93.7)	N/A	189 (95.0)	63 (86.3)	N/A
Severity of illness >0^4^	(Yes)	116 (22.4)	47 (24.6)	14 (18.9)	N/A	47 (25.0)	8 (12.7)	N/A

1 Includes 17 patients from the medical intensive care unit.

2 Includes five patients from the surgical intensive care unit.

3 Comorbidity due to chronic illnesses such as kidney-related diseases, hypertension, diabetes, asthma plus pneumonia, frequent dizziness, ulcers, dementia, liver disease, connective tissue disease, etc.

4 Severity of illness based on clinical assessment using the McCabe score for underlying illness: (0 = normal life expectancy; 1 = ultimately fatal life expectancy <5 years; and 2 = rapidly fatal life expectancy <1 year). NA—not applicable to patients in the uninfected cohort because the validated instrument, adapted from the WHO study on AMR-attributable mortality, made it conditional and applicable to only patients with bloodstream infections/AMR. NICU, neonatal intensive care unit; PICU, paediatric intensive care unit.

For clear presentation and emphasis on AMR-attributable LOS and provider cost, the subsequent sections first compare the study outcomes between the AMR and susceptible cohorts, followed by a summary comparison between the AMR and uninfected cohorts, as shown in all result tables.

### Hospital resource used

In 2021, there were 35 492 hospital admissions at Hospital 1 and 49 138 at Hospital 2. The reported mean LOS obtained from the hospitals’ annual report was 7.6 days at Hospital 1 and 8.9 days at Hospital 2. In contrast, the estimated LOS for the select groups of patients in this study was considerably higher (Table 2). For instance, the estimated LOS at Hospital 1 was 16.5 days (95% CI: 15.8–17.3) for patients in the AMR cohort and 12.3 days (95% CI: 11.4–13.1) for the susceptible cohort. The stratified analysis indicates that the mean extra LOS due to AMR if compared to the susceptible cohort was 5.9 days (95% CI: 3.5–8.3) for MRSA-infected patients, 4.7 days (95% CI: 1.7–7.7) for *E. coli*-infected patients and 3.9 (95% CI: 1.5–6.3) for *Klebsiella pneumoniae*-infected patients. Compared to the susceptible cohort, AMR patients consumed an extra 434 and 202 minutes of doctor’s time at Hospitals 1 and 2, respectively (Table 2).

**Table 2.** Hospital resource use indicators (2021)

	Hospital 1	Hospital 2	Overall
Hospital-level indicator			
Mean LOS^∞^	7.6	8.9	8.2
Mean LOS (95% CI) by patient cohort^1^			
Mean LOS for the overall sample	12.8 (12.3–13.3)	14.3 (13.7–14.9)	13.5 (13.1–13.9)
Mean LOS for Uninfected cohort	9.2 (8.6–9.7)	10.3 (9.6–10.9)	9.7 (9.3–10.1)
Mean LOS for Susceptible cohort	12.3 (11.4–13.1)	13.2 (12.2–14.2)	12.7 (12.1–13.4)
Mean LOS for AMR cohort	16.5 (15.8–17.3)	18.7 (17.8–19.6)	17.6 (17.0–18.2)
Mean extra LOS due to AMR bacterial type compared with the susceptible cohort^1^			
*E. coli*	4.2 (1.1–7.3)	5.1 (2.2–8.1)	4.7 (1.7–7.7)
*K. pneumoniae*	3.3 (1.1–5.5)	4.5 (2.0–7.1)	3.9 (1.5–6.3)
*S. aureus* (MRSA)	5.0 (3.1–6.9)	6.7 (3.8–9.7)	5.9 (3.5–8.3)
Mean extra staff time^#^ used due to AMR compared with the susceptible cohort^1^			
Overall (AMR cohort)	434** (267–600)	202* (41–363)	317** (201–433)
*E. coli*-infected patient	396* (85–707)	214 (−28–457)	287** (99–475)
*K. pneumoniae*-infected patients	343* (70–616)	54 (−176–283)	191* (15–368)
*S. aureus*-infected patients	517** (243–791)	356 (−52–764)	453** (226–680)

∞ Obtained from participating hospital’s annual reports 2021.

1 Primary data.

# Time in minutes.

* <0.5, **< 0.01.

### Healthcare provider expenditures

In 2021, the total expenditure for both hospitals amounted to approximately $128 million. Expenses on goods and services, including medical supplies, utilities and maintenance equalled 66% of the total annual expenditure. Staff compensation and salaries excluded US$3.48 million meant to incentivize coronavirus disease 2019 (COVID-19) frontline workers from July to September 2021. We excluded the incidental cost of US$3.48 million because it does not constitute the usual recurrent expenditure, and there were administrative and accountability issues related to COVID-19 expenditures in both hospitals. Using the mean LOS reported by each hospital, the estimated total bed days at Hospitals 1 and 2 were 269 739 and 437 328, respectively. Dividing the total annual expenditure by the total hospital bed days yielded an average of US$196 and US$172 HPCs of caring for inpatients per bed day at Hospitals 1 and 2, respectively (Table 3).

**Table 3.** Hospital expenditures in US$ (2021 PPP-adjusted)

Description	Hospital 1	Hospital 2	Overall
Variable cost			
Staff compensation/salaries	10 880 757	16 965 085	27 845 842
Goods and services	36 098 127	49 171 246	85 269 373
Sub-total	46 978 884	66 136 331	113 115 215
Fixed cost			
Assets (annualized)	5 810 643	9 192 829	15 003 472
Grand total expenditure	52 789 527	75 329 160	128 118 687
Total bed days*	269 739	437 328	707 067
HPC per day	196	172	181

Source: Hospital annual reports, 2021.

* Multiplying the hospital-reported mean annual LOS by the total admissions in 2021.

### HPCs associated with AMR

In Table 4, the estimated provider cost associated with AMR if compared to the susceptible cohort corresponds to US$823 (95% CI: US$812–US$863) at Hospital 1 and US$946 (95% CI: US$929–US$964) at Hospital 2. The estimate is equivalent to US$653 423 annually (95% CI: US$643 117–US$675 081) in total for both hospitals.

**Table 4.** Estimated additional HPC due to AMR in US$ (2021 PPP-adjusted)

	Case 1. AMR cohort compared to susceptible cohort				Case 2. AMR cohort compared to uninfected cohort		
	AMR cohort	Case 1. AMR cohort compared to susceptible cohort			Case 2. AMR cohort compared to uninfected cohort		
	**Mean HPC, AMR cohort (95% CI)**	**Mean HPC, susceptible cohort (95% CI)**	**Difference from AMR cohort**	Estimated annual HPC due to AMR*	**Mean HPC, uninfected cohort (95% CI)**	**Difference from AMR cohort**	Estimated annual HPC due to AMR**
Hospital 1	3234 (3097–3391)	2411 (2234–2568)	823 (812–863)	311 917	1803 (1686–1901)	1432 (1411–1490)	542 728
Hospital 2	3216 (3062–3371)	2270 (2098–2442)	946 (929–964)	341 506	1772 (1651–1875)	1444 (1411–1496)	521 284
Total				653 423			1 064 012
**Adjusted mean extra provider cost of AMR for surviving patients in US$ (2021 PPP-adjusted)**							
		**Case 1. AMR cohort compared to susceptible cohort**			**Case 2. AMR cohort compared to uninfected cohort**		
		**Mean HPC per patient due to AMR**	**Mean total HPC due to AMR^1^**	Estimated annual HPC due to AMR*	**Mean HPC per patient due to AMR**	**Mean total HPC due to AMR^1^**	Estimated annual HPC due to AMR**
Hospital 1		883 (627–1137)	155 408	334 657	1471 (1254–1627)	258 896	557 509
Hospital 2		975 (698–1204)	161 850	351 975	1645 (1473–1734)	273 070	593 845
Total				686 632			1 151 354

* Compared to the susceptible cohort, **compared to the uninfected cohort.

Based on the observed AMR prevalence of 6.9% at Hospital 1, 8.0% at Hospital 2 and 7.4% at both sites combined, equivalent to 740 annual AMR cases due to MRSA and 3GC-resistant *K. pneumoniae* and *E. coli* bloodstream infections.

### Adjusted cost for surviving patients with AMR

Among AMR patients who survived the 30 days of follow-up (i.e. Hospital 1, *n* = 176; Hospital 2, *n* = 166), the estimated mean difference in the costs due to AMR was US$883 (95% CI: US$627–US$1137) at Hospital 1 and 975 (95% CI: US$698–US$1204) at Hospital 2 if compared to the susceptible cohort. Relative to the unadjusted costs, the estimated annual provider cost due to AMR at Hospitals 1 and 2 was US$22 740 and US$10 469 for the study period, equivalent to US$188 429 extra cost annually in both hospitals combined (Table 4).

### Results comparing the crude costs with step-down costs

Table 5 illustrates the results comparing the crude cost estimates with the more precise step-down costs for each unit/department where AMR patients received treatment. Compared to the crude estimate, the HPC associated with AMR, using step-down cost allocation, was 15% and 0.1% less at Hospital 1 medical and paediatric units, respectively, where we recorded more cases of AMR. However, the cost was 141% and 41% higher at the medical emergency and maternity units, respectively, where few cases of AMR were recorded. In the same vein, the cost was 36% and 8% less at Hospital 2 medical and paediatric units but 115% and 31% high at the emergency and maternity units, respectively. Overall, there was a 9% increase in the estimated mean annual HPCs at Hospital 1 and a 16% decrease at Hospital 2 (Table S6).

**Table 5.** A comparison of the cost centre estimates of the HPCs of AMR in US dollars (2021 PPP-adjusted)

	Hospital 1			Hospital 2		
	**Estimated mean additional HPC due to AMR (95% CI)**			**Estimated mean additional HPC due to AMR (95% CI)**		
**Cost centres**	**Crude cost** **(primary case**)	**Step-down cost** **(secondary case)**	**% Change**	**Crude cost** **(primary case)**	**Step-down cost** **(secondary case)**	**% Change**
Medical	764 (372–1137)	652 (318–970)	−15%	1152 (688–1600)	737 (560–1301)	−36%
Paediatric	843 (353–1352)	842 (352–1351)	−0.1%	877 (413–1342)	806 (379–1232)	−8%
PICU				688 (189–1565)	696 (191–1583)	−1%
NICU				1256 (52–2477)	1302 (54–2569)	4%
Maternity*	549 (235–862)	776 (332–1219)	41%	430 (155–705)	565 (203–926)	+31%
Emergency*	1000 (294–1705)	2409 (708–4109)	141%	464 (−688–1617)	998 (−1478–3473)	+115%
Surgical*	1058 (470–1646)	681 (303–1059)	−36%	1032 (−1049–3113)	881 (−895–2658)	−15%

Note. NICU, neonatal intensive care unit; PICU, paediatric intensive care unit.

* Few cases of AMR leading to wider 95% CIs.

### HPC of AMR by bacterial type

Compared to the susceptible cohort, the mean extra HPC due to AMR at Hospital 1 was US$980 (95% CI: US$608–US$1352) for patients with MRSA bloodstream infections and US$823 (95% CI: US$216–US$1431) and US$647 (95% CI: US$227–US$1078) for BSI patients with *E. coli* and *K. pneumoniae* infections resistant to 3GC, respectively. In the same order, the cost was US$1152 (95% CI: US$654–1668), US$877 (95% CI: US$378–US$1393) and US$774 (US$344–US$1221) at Hospital 2, respectively (Table 6). While the estimated annual cost of *E. coli* patients is ∼30% of costs, the two hospitals differ in the sense that *K. pneumoniae* cases account for only 17% of costs at Hospital 1 but 37% at Hospital 2. Thus, despite a slightly lower cost per patient, the high number of MRSA cases at Hospital 1 contributed to increased total costs for MRSA patients compared to the estimated total cost for MRSA patients at Hospital 2.

**Table 6.** Mean HPC due to AMR by bacteria type in US$ (2021 PPP-adjusted)

	Hospital 1			Hospital 2		
**AMR causative pathogen**	**Mean^1^ HPC due to AMR** **(95% CI)**	**Mean** **total** **HPC due to AMR**	Estimated annual HPC due to AMR*	**Mean^1^ HPC due to AMR** **(95% CI)**	**Mean** **Total** **HPC due to AMR**	Estimated annual HPC due to AMR*
*S. aureus (*MRSA)	980 (608–1352)	90 160	175 420	1152 (654–1668)	63 360	112 896
*E. coli*	823 (216–1431)	39 504	93 822	877 (378–1393)	75 422	92 085
*K. pneumoniae*	647 (227–1078)	42 055	55 642	774 (344–1221)	44 892	122 292
Total			324 884			327 273

1 Based on cost difference between AMR cohort and susceptible cohort.

### Results comparing the provider cost between AMR and uninfected cohorts

Between the AMR and uninfected cohorts, the estimated mean difference in provider cost was US$1432 (95% CI: US$1411–US$1490) at Hospital 1 and US$1444 (95% CI: US$1411–1496), indicating that the cost was comparable between the two hospitals. In total, the magnitude of the potential annual provider cost savings without bloodstream infection is almost double compared to having susceptible AMR infection.

## Discussion

Using both aggregated ingredient costing and step-down costing approaches, this study quantified the HPCs due to AMR at two public tertiary hospitals in Ghana, a lower-middle-income setting. The result shows that healthcare providers spent significantly longer time attending to AMR patients than patients infected with susceptible bacteria and uninfected patients. For example, the findings suggest that if AMR is avoided, the extra 5 bed days allocated for treating an estimated 740 AMR patients annually at both hospitals would release 3700 bed days to admit extra 451 patients. Again, doctors spend approximately an extra 7 hours at Hospital 1 and 3 hours at Hospital 2 caring for each AMR patient admitted to the hospital within the 30 days duration of the data collection. Therefore, in the absence of AMR, doctors would have more time at their disposal to attend to other critically ill patients.

The crude analysis indicated that the extra bed days due to AMR correspond to increases in HPCs, which vary between causative organisms, surviving patients and cost centres where AMR patients received treatment. For instance, the provider cost of treating each patient with MRSA bloodstream infections at Hospital 1 was ∼33% higher than *K. pneumoniae* and 16% higher than *E. coli*bloodstream infections resistant to 3GC. In the same manner, the cost was ∼33% and 23% high at Hospital 2. This noticeable variation in cost due to AMR causative pathogens has implications for healthcare providers regarding where to focus interventions. As it is difficult to prioritize AMR intervention at hospitals by focusing on specific bacteria, we argue that general infection prevention and control and effective antibiotic regulation by providers and policymakers may be useful to counter the spread of AMR. Additionally, by adhering to WHO’s access, watch and reserve (AWaRe) recommendation for antibiotic prescription and therapy (World Health Organization, 2021), healthcare providers can help reduce the risk of MRSA and other resistant bacterial infections, which are more costly to treat. We argue that adherence to AWaRe prescribing practices by providers should consider local context like national resistance patterns. Also, the estimated annual provider cost attributable to AMR was ∼11% higher for surviving patients that completed the 30-day follow-up period because they consumed more hospital resources than those who died before the end of the follow-up. By implications, the increased use of limited hospital resources and the corresponding provider costs of treating surviving AMR patients are other reasons why healthcare providers and policymakers should prioritize AMR mitigation measures.

The secondary analysis showed that the estimated mean HPC due to AMR was sensitive to the cost allocation parameters, including staffing, consumables and fixed assets. In both hospitals, the provider cost of AMR was highest for the medical emergency unit because the unit is resourced but has limited capacity to treat patients with severe and emergency health conditions compared to the general medical unit where many patients with internal medical conditions such as diabetes, stroke, hypertension and other mild-to-moderate health symptoms are treated. For instance, the total bed days at the emergency unit in 2021 equalled 8.7% and 14% of the total bed days at the general medical unit at Hospitals 1 and 2, respectively. In general, comparing the step-down estimated costs with the crude estimated costs showed that the former approach underestimated the provider cost of AMR at Hospital 1 by 9% and overestimated the same by 16% at Hospital 2. This implies that using both costing approaches, the estimated annual AMR-attributable provider costs are much closer (crude estimate: US$686 632 versus step-down estimate: US$637 890).

The findings from this study indicate that for the two hospitals, the provider cost associated with AMR amounts to $650 000 a year for treating only three AMR organisms, that is, MRSA*, E. coli* and *K. pneumoniae* resistant to 3GC. Therefore, the actual costs of AMR are higher due to other AMR pathogens if other antibiotic-resistant organisms like *Enterobacter* spp., *Citrobacter* spp., *Acinetobacter* spp. *and Pseudomonas aeruginosa* were included in this study.

Based on these results, we argue that there is a strong economic incentive for policymakers and healthcare providers, especially in LMIC settings to invest time and resources in AMR mitigation strategies to reduce the spread of AMR and its associated HPCs. For providers, AMR mitigation strategies may focus on the demand or supply of antibiotics. Demand-driven strategies may include point-of-care education regarding antibiotic use and resistance’s health and economic implications, as discussed in a previous study (Otieku *et al.*, 2023b). Supply-driven interventions may consider hospital-wide antibiotic restriction policy implementation as shown to reduce antibiotic consumption by 42% and their costs by 31% in some European countries (Antoniadou *et al.*, 2013; Ntagiopoulos *et al.*, 2017). We also see a strong need for policies and politicians to promote a regulatory regime regarding the sale of antibiotics to consumers by pharmacies and other retailers and to the veterinary and food production sectors. Other generic cost-effective interventions may include one that prevents bacterial infections, such as multimodal hand hygiene using alcohol-based hand-rub and frequent washing of hands with soap and water (Saharman *et al.*, 2021; Allegranzi *et al.*, 2013; ), as well as infection surveillance and AMR stewardship (Seale *et al.*, 2017; Maki and Zervos, 2021; Otieku *et al.*, 2022). For example, effective AMR surveillance systems in hospitals and quicker diagnostic test in communities will enhance identification and planned intervention strategies to address national prevalence of AMR (Okolie *et al.*, 2022).

The strength of this study can be attributed to the use of reliable data and cost estimation procedures aimed at generalization and precision costing for policymaking. For example, the primary analysis considered the overall hospital expenditure quantified by the hospitals and obtained from their annual financial report, which warrants internal validity of the results. Again, the methodology considered standard costing approaching for external validity. Studies reporting the provider costs of AMR in LMIC settings are scarce. Therefore, this study makes a unique contribution to the literature. We believe in settings like Ghana where hospital resources are scarce, and the finding will serve the intended purpose of helping healthcare providers to decide on the cost-benefit of AMR interventions and at the same time support Ghana’s policy on antimicrobial use and resistance.

Regarding limitations, we could not obtain data on nurses’ time consumed from patients’ folders in the same way as recorded for doctors resulting in an underestimation of staff time consumed. Again, although the step-down cost allocation considered that the burden of resources used per bed day was not the same across departments, we still applied average estimates to quantify the departmental cost of AMR, which may lead to an underestimation of the cost because we expect AMR patients to be more resource demanding than the average patient without AMR infection. From the perspective of the hospitals, some staff were not on the payroll of the hospitals because they were from institutions outside Ghana undergoing training or collaborating on specific projects for a short duration. Therefore, data on the overhead costs of staff from other institutions were not captured in the personnel costs, leading to an underestimation of the endpoint costs. Also, though rotation staff in Hospital 2 performed essential clinical services at multiple clinical units, their cost was captured only once to avoid overestimation of the personnel cost. This was necessary because the rotation was within the same department, and they were not paid more for the multiple rotations. The best approach to estimate the cost of rotation staff was to consider their time allocation to other units, but such data was unavailable at the time of data collection. Again, due to data limitation, we could not distribute the annual expenditure by the laboratory service directorate using the best approach, which requires that we divide the cost by the actual number of patients utilizing the laboratory service across the departments. The alternative was to divide the expenditure by the laboratory service directorate equally for all departments based on expert opinion at the laboratory, which implies that we may underestimate or overestimate the endpoint cost for some units. Furthermore, the imperfect age criterion for matching contributed to a situation where a few matched patients (*n* = 11) in the AMR cohort had more severe diseases than their matches in the susceptible and uninfected groups. Also, patient admissions during the COVID-19 pandemic (2020 and 2021) reduced by 17% in Hospital 1 and 21% in Hospital 2 compared to the pre-pandemic period (2018 and 2019), and this contributed to reduced hospital expenditures on annual consumables and underestimation of the endpoint provider cost attributable to AMR. Our results mostly compare outcomes between the AMR and susceptible cohorts for clear presentation and emphasis on AMR-attributable cost. However, we have also shown that the potential AMR-attributable cost saving is almost double when comparing with similar patients without bloodstream infection. This is important since many interventions to prevent AMR infectious will also help reduce BSI altogether.

## Conclusion

AMR imposes significant costs on healthcare providers in LMIC settings. This cost is avoidable through a myriad of infection prevention and control strategies, including effective community and hospital AMR stewardship, surveillance/diagnostics and public awareness campaigns.

## Supplementary Material

czad114_Supp

## Data Availability

The data used for this study are publicly available and can also be obtained from the corresponding author.

## Reflexivity statement

This study is part of a 3-year doctoral research project on the economic analysis of AMR in a lower-middle-income setting, of which the corresponding author was a PhD student and supervised by U.E., J.A.L.K. and A.P.F., while A-K.L. and A.O.O. were collaborators from the participating hospitals in Ghana. Therefore, the authors comprise a multidisciplinary team from Ghana and Denmark with expertise in health economics, microbiology and infectious diseases, public health and health system research.
