# Supplementary material for: Healthcare provider cost of antimicrobial resistance in two teaching hospitals in Ghana
Source: Health Policy Plan. 2023 Dec 4;39(2):178–87. doi: 10.1093/heapol/czad114 (PMC11020270; doi:10.1093/heapol/czad114)
Supplement: czad114_Supp [file czad114_supp.zip › suppl_data/Supplementary file.docx]

**Supplementary material**

**Table S1. Staff distribution, bed capacity, and total admissions by hospital**

|  | **Hospital 1** | **Hospital 2** | **Total staff** | **The assumption for personnel cost allocation in the primary analysis (base case)** |
| --- | --- | --- | --- | --- |
| Management staff | 16 | 21 | 37 | Assuming all types of patients (AMR and non-AMR) demanded resources equally, requiring that we apply average resource use for quantification. |
| Medical staff |  |  |  |  |
| Physicians & physician consultants | 413 | 538 | 951 |  |
| Nurses & midwives | 2,186 | 3,272 | 5,458 |  |
| Others^1^ | 724 | 1,006 | 1,730 |  |
| Non-medical staff^2^ | 707 | 927 | 1,634 |  |
| Total number of staff | 4,046 | 5,764 | 9,810 |  |
| Bed capacity | 1200 | 2000 |  |  |
| Admissions | 35,492 | 49,138 |  |  |
| OPD attendance | 316,287 | 453,862 |  |  |

^1^Includes certified registered anaesthetists, allied health staff, pharmacists, and other clinical support

^2^Includes Finance, audit, general administrative clerks, and auxiliary staff.

**Note**. Being a tertiary hospital, about 40% of physicians are resident and senior resident doctors and about a third of nurses are under training.

Abbreviations: AMR – antimicrobial resistance, OPD – outpatient department.

**Table S2. Cost items, data source, and assumptions for cost allocation factor in a secondary analysis.**

| **Cost items** | **Data sources** | **Cost calculation assumption** |
| --- | --- | --- |
| **Step 1. Unit/departmental specific cost allocation considerations** | | |
|  | **Capital costs** | |
| Building size and quantity | Hospital annual financial report and asset valuation register | The number and size of buildings owned per department or cost centres where AMR patients received treatment were calculated per square meter (m^2^) and multiplied by the annualised unit costs*. |
| Other assets (equipment, furniture, vehicles) | Hospital annual financial report and asset valuation register | The quantity of items used by each cost centre is multiplied by the annualised unit costs*. |
|  | **Recurrent Costs** | |
| Personnel | Departmental duty roster and hospital annual report | The number of staff rendering clinical, administrative, and auxiliary services times the average unit cost of personnel in each cadre/speciality at the cost centre.  For rotation staff in hospital 2, we did not have information on time allocation between units. Therefore, we assumed either rotation to other units is negligible (zero), or time allocation nets out (zero). |
| Medical supplies (drugs, protective gear, disposables, etc.) | Procurement records | The quantity of medical supplies consumed by the cost centre times their unit costs. That means purchased items not used during the 2021 financial year were excluded. |
| Non-medical supplies (toiletries, stationary, data, electricity, water, etc) | Procurement records | The quantity of non-medical supplies consumed by the cost centre times their unit costs. That means purchased items not used during the 2021 financial year were excluded. |
| Other recurrent costs (cleaning, laundry, medical waste management, catering, periodic maintenance, etc.) | Finance and budget records | The monthly unit charges were multiplied by the number of months services were consumed by each cost centre in 2021**.** |
| **Step 2. Shared cost allocation to cost centres** | | |
|  | | |
| Laboratory Service Directorate. | Hospital annual report, asset register, and unit administration records. | The total annual cost incurred by the laboratory service directorate for the costing year considered both recurrent and annualised capital costs.  We considered two approaches for distributing shared cost. The best approach is dividing the total cost/expenditure using the actual patient ratio/proportion utilizing the laboratory/diagnostic service across the department as one will anticipate that the departments vary in terms of bed capacity and resource consumption. However, data on actual resource used (laboratory service used) was unavailable at the time of data collection, but interview with the laboratory administrators indicate no significant difference in workload burden imposed by different departments. This is because smaller units like neonatal intensive care unit have small bed capacity, but the consumption of laboratory services per patient is over 10 times more than patients in bigger units like the general medical ward. Based on the staff perceptions, a crude alternative was to distribute the cost equally between clinical departments. |

*The valuation is done yearly by specialized valuers contracted by the hospitals and the annual costs are reported in the hospital’s annual financial report.

**Note.** If two or more units/departments occupy one building complex, the cost of utilities is shared based on the average consumption size obtained from billing and expenditure records.

**Table S3. Allocation factor for step-down costing (Secondary analysis)**

|  | **Hospital 1** | | | | **Hospital 2** | | | |
| --- | --- | --- | --- | --- | --- | --- | --- | --- |
| **Department/Directorate/Unit** | **Staff** | **Admissions** | **Mean LOS**  **[95%CI]** | **OPD attendance** | **Staff** | **Admissions** | **Mean LOS**  **[95%CI]** | **OPD attendance** |
| Maternity^1^ | 429 | 9,488 | 4.7 [4.5 – 4.9] | 41,312 | 611 | 12,383 | 5.8 [5.7 – 5.9] | 75,313 |
| Medical (Emergency Medicine) | 311 | 1,041 | 4.2 [3.9 – 4.5] | N/A | 481 | 2,015 | 4.9 [4.6 – 5.2] | N/A |
| Medical (Internal Medicine) | 571 | 4,978 | 10.1 [9.8 – 10.4] | 53,697 | 698 | 5,717 | 12.2 [12.0 – 12.4] | 58,766 |
| Surgical^2^ (General surgical unit) | 398 | 4,617 | 12.4 [12.2 – 12.6] | 7,324 | 577 | 3,062 | 11.7 [11.3 – 12.1] | 13,926 |
| *Neonatal Intensive Care Unit | - | - | - |  | 102 | 2,171 | 11.5 [11.1 – 11.9] | 4,137 |
| *Paediatric | 248 | 3,169 | 7.1 [7.0 – 7.2] | 16,565 | 391 | 5,016 | 9.2 [8.6 – 9.8] | 22,325 |
| *Paediatric intensive care unit | - | - | - |  | 101 | 977 | 8.5 [7.3 – 9.7] | 1,429 |
| Total | 2,094 |  |  |  | 2,961 |  |  |  |

**Source.** Hospital annual report and Biostatistics Unit, 2021

^1^Exclude staffing and admissions to gynaecology and reproductive health units, ^2^Exclude staff and admissions to Neurosurgery, Paediatric surgery, Urology and Allied surgery (dental, eye, ear, nose, throat).

*All staff are under the Department of Child Health and allocations of medical staff are based on speciality.

**Note.** Staffing considered the actual number of personnel providing direct and indirect care such as treatment, dispensary, surveillance, and domestic services, that is, cleaning, laundry, catering, etc. About 85% of the staff provide direct treatment care to patients.

Abbreviations: LOS – length of stay, N/A – not applicable, OPD – outpatient department.

**Table S4. Categories of annual expenditure stratified by costs centres per hospital in US$ (2021 PPP adjusted)**

|  | **Hospital 1** | | | | **Hospital 2** | | | |
| --- | --- | --- | --- | --- | --- | --- | --- | --- |
| **Cost centres** | **Staff** | **Consumables** | **Annualized asset** | **Total** | **Staff** | **Consumables** | **Annualized asset** | **Total** |
| Maternity | 1,153,123.71 | 9,645,966.32 | 1,553,346.69 | 12,352,436.72 | 1,520,728.20 | 12,391,378.14 | 2,316,634.81 | 16,228,741.15 |
| Medical (Emergency Medicine) | 835,947.50 | 1,058,332.65 | 170,432.52 | 2,064,712.67 | 1,254,615.82 | 2,016,370.20 | 376,966.20 | 3,647,952.22 |
| Medical (Internal Medicine) | 2,534,810.36 | 5,060,883.70 | 814,998.16 | 8,410,692.22 | 2,965,741.87 | 5,720,887.56 | 1,069,536.36 | 9,756,165.79 |
| Surgical (General surgical unit) | 1,769,797.73 | 4,693,873.05 | 755,895.24 | 7,219,566.02 | 1,624,975.73 | 3,063,929.06 | 572,838.96 | 5,261,743.75 |
| Neonatal Intensive Care Unit | - | - | - | - | 1,875,257.41 | 2,172,476.28 | 406,150.68 | 4,453,884.37 |
| Paediatric | 666,607.63 | 3,221,763.85 | 518,828.68 | 4,407,200.16 | 1,332,010.50 | 5,019,410.88 | 938,393.28 | 7,289,814.66 |
| Paediatric intensive care unit | - | - | - | - | 284,441.16 | 977,664.36 | 182,777.16 | 1,444,882.68 |

**Note.** The cost of staffing excludes those on the payroll of affiliate Universities and other local and international institutions rendering support services at the hospitals.

**Table S5. Estimated LOS due to AMR and the overall hospital bed days stratified by cost centres per hospital**

|  | **Hospital 1** | | |  | **Hospital 2** | | |
| --- | --- | --- | --- | --- | --- | --- | --- |
| **Unit** | **Estimated LOS due to AMR [95% CI]** | **Estimated total bed days** | **Estimated annual cases of AMR** |  | **Estimated LOS due to AMR [95% CI]** | **Estimated total bed days** | **Estimated annual cases of AMR** |
| Maternity | 2.8 [1.2 – 4.4] | 44,594 | 5 |  | 2.5 [0.9 – 4.1] | 71,821 | 4 |
| Emergency Medicine | 5.1 [1.5 – 8.7] | 4,372 | 46 |  | 2.7 [-4 – 9.4] | 9,874 | 28 |
| Internal Medicine | 3.9 [1.9 – 5.8] | 50,278 | 198 |  | 6.7 [4.0 – 9.3] | 69,747 | 146 |
| Surgical | 5.4 [2.4 – 8.4] | 57,251 | 43 |  | 6 [-6.1 – 18.1] | 35,825 | 15 |
| Neonatal Intensive Care Unit | - | - | - |  | 7.3 [0.3 – 14.4] | 24,967 | 19 |
| Paediatric | 4.3 [1.8 – 6.9] | 22,500 | 87 |  | 5.1 [2.4 – 7.8] | 46,147 | 110 |
| Paediatric intensive care unit | - | - | - |  | 4.0 [-1.1 – 9.1] | 8,305 | 39 |

Abbreviation. AMR – antimicrobial resistance, LOS – length of stay

**Table S6. A comparison of the cost centre estimates of the healthcare provider costs of AMR in US dollars (2021 PPP adjusted).**

|  | **Hospital 1** | | | | **Hospital 2** | | | |
| --- | --- | --- | --- | --- | --- | --- | --- | --- |
| Cost Centres | Estimated mean additional healthcare provider cost due to AMR [95%CI] | | Estimated mean annual healthcare provider cost due to AMR  (% change) | | Estimated mean additional healthcare provider cost due to AMR [95%CI] | | Estimated mean annual healthcare provider cost due to AMR  (% change) | |
|  | Crude cost  [Primary case] | Step-down cost  [Secondary case] | Crude cost  [Primary case] | Step-down cost  [Secondary case] | Crude cost  [Primary case] | Step-down cost  [Secondary case] | Crude cost  [Primary case] | Step-down cost  [Secondary case] |
| Medical | 764  [372 – 1,137] | 652  [318 – 970] | 151,272 | 129,096 (-15%) | 1,152  [688 – 1,600] | 737  [560 – 1,301] | 168,192 | 107,602 (-36%) |
| Paediatric | 843  [353 – 1,352] | 842  [352 – 1351] | 73,341 | 73,254 (-0.1%) | 877  [413 – 1,342] | 806  [379 – 1,232] | 96,470 | 88,660 (-8%) |
| PICU | - | - | - | - | 688  [189 – 1,565] | 696  [191 – 1,583] | 26,832 | 27,144 (-1%) |
| NICU | - | - | - | - | 1,256  [52 – 2,477] | 1,302  [54 – 2569] | 23,864 | 24,738 (+4%) |
| Maternity* | 549  [235 – 862] | 776  [332 – 1,219] | 2,745 | 3,880 (+41%) | 430  [155 – 705] | 565  [203 – 926] | 1,720 | 2,260 (+31%) |
| Emergency* | 1000  [294 – 1,705] | 2,409  [708 – 4,109] | 46,000 | 110,814 (+141%) | 464  [-688 – 1,617] | 998  [-1478 – 3473] | 12,992 | 27,944 (+115%) |
| Surgical* | 1058  [470 – 1,646] | 681  [303 – 1059] | 45,494 | 29,283 (-36%) | 1,032  [-1,049 – 3,113] | 881  [-895 – 2,658] | 15,480 | 13,215 (-15%) |
|  |  |  | 318,852 | 346,327 (+9%) |  |  | 345,550 | 291,563 (-16%) |

**Note.** NICU – neonatal intensive care unit. PICU – paediatric intensive care unit.

*Few cases of AMR leading to wider 95% confidence intervals.
